# Burdens of Invasive Methicillin-Susceptible and Methicillin-Resistant *Staphylococcus aureus* Disease, Minnesota, USA

**DOI:** 10.3201/eid2501.181146

**Published:** 2019-01

**Authors:** Mackenzie Koeck, Kathryn Como-Sabetti, Dave Boxrud, Ginette Dobbins, Anita Glennen, Melissa Anacker, Selina Jawahir, Isaac See, Ruth Lynfield

**Affiliations:** Minnesota Department of Health, St. Paul, Minnesota, USA (M. Koeck, K. Como-Sabetti, D. Boxrud, G. Dobbins, A. Glennen, M. Anacker, S. Jawahir, R. Lynfield);; Centers for Disease Control and Prevention, Atlanta, Georgia, USA (I. See)

**Keywords:** Staphylococcus aureus, bacteria, methicillin-susceptible S. aureus, methicillin-resistant S. aureus, MSSA, MRSA, antimicrobial resistance, invasive disease, disease burden, Minnesota, United States

## Abstract

During August 1, 2014–July 31, 2015, in 2 counties in Minnesota, USA, incidence of invasive methicillin-susceptible *Staphylococcus aureus* (MSSA) (27.1 cases/100,000 persons) was twice that of invasive methicillin-resistant *S. aureus* (13.1 cases/100,000 persons). MSSA isolates were more genetically diverse and susceptible to more antimicrobial drugs than methicillin-resistant *S. aureus* isolates.

Methicillin-resistant *Staphylococcus aureus* (MRSA) infections were first reported in the 1960s in healthcare facilities (*1*). Risk factors included recent hospitalization, surgery, dialysis, having a central venous catheter or other invasive medical device, chronic wounds, residence in long-term care facilities or prisons, injection drug use, and exposure to antimicrobial drugs (*2*). In the 1990s, MRSA infections caused by genetically distinct strains were observed among healthy persons in the community (*3*).

Although *S. aureus* has long been recognized as a major human pathogen, epidemiologic studies and infection prevention precautions in recent decades have largely focused on MRSA. We compared epidemiologic, microbiologic, and molecular characteristics of invasive methicillin-susceptible *S. aureus* (MSSA) infections with those of invasive MRSA infections.

## The Study

Active surveillance for invasive MSSA was added to existing invasive MRSA surveillance in Ramsey and Hennepin Counties (combined population in 2014: 1,742,806) in Minnesota, USA, during 2014. Cases were defined as *S. aureus* isolated from a normally sterile site in a catchment area resident. We collected demographic and clinical information from medical record reviews by using a standardized case report form. We epidemiologically characterized patients with invasive *S. aureus*: hospital onset (culture obtained >3 days after admission); healthcare-associated, community onset (HACO; >1 healthcare-associated risk factor); and community associated (CA). Healthcare-associated risk factors included in the year before culture hospital admission or residence in a long-term care facility or long-term acute-care facility for any duration, surgery, or dialysis or within 2 days before culture presence of a central vascular catheter.

Invasive MSSA and MRSA isolates were submitted to the Minnesota Department of Health for species confirmation, antimicrobial drug susceptibility testing, and molecular characterization. We confirmed species by using the tube coagulase test (Becton Dickinson, http://www.bd.com/) or matrix-assisted laser desorption ionization time-of-flight mass spectrometry by using a Microflex LT/SH mass spectrometer and FlexControl 3.4 software (Bruker Daltonics, Inc., https://www.bruker.com/). We subtyped isolates by using pulsed-field gel electrophoresis (PFGE) according to published protocols (*4*). PFGE patterns were analyzed by using BioNumerics software (Applied Maths, http://www.applied-maths.com/); patterns without differences were considered indistinguishable. We assigned patterns a USA clonal group on the basis of an 80% similarity coefficient cutoff to a type strain. Whole-genome sequencing characterized 2 isolates that were untypable by PFGE. We determined sequence type by using short reads in Illumina Miseq as described (*5*) and retrieved sequences by uploading raw read files to the Multilocus Sequence Typing 1.8 server (*S. aureus* configuration) hosted by the Center for Genomic Epidemiology (*6*).

We performed antimicrobial drug susceptibility testing by using broth microdilution with a custom dried panel (TREK Diagnostic Systems, Inc., http://www.trekds.com/techInfoVT/default.asp) and interpreted results according to Clinical and Laboratory Standards Institute guidelines (*7*). Isolates susceptible to penicillin were examined for β-lactamase production by using nitrocefin disk test, penicillin zone-edge test, and *blaZ* β-lactamase gene confirmation by PCR. We induced β-lactamase production for nitrocefin disk testing by inoculating an isolate onto sheep blood agar with a 1-μg oxacillin disk and incubating overnight by using TREK Diagnostic Systems. Subsequent growth from the zone periphery was incubated with a nitrocefin disk (REMEL, http://www.remel.com/About.aspx) for 60 min. We also performed a penicillin zone-edge test by using standard disk diffusion, followed by assessment for fuzzy or sharp zone edges. We determined the incidence of the *blaZ* β-lactamase gene by using real-time PCR as described but with slight modifications (*8*).

We analyzed data by using SAS version 9.4 (SAS Institute, https://www.sas.com). Census data for 2014 were used to calculate incidence. We used a Wald χ^2^ test and p<0.05 to distinguish statistical significance. Infections with invasive MSSA and MRSA are reportable conditions under Minnesota State Rules, and obtaining information for surveillance purposes does not require Institutional Review Board review.

A total of 701 cases (473 invasive MSSA, 228 invasive MRSA) were reported during August 1, 2014–July 31, 2015. Incidence for invasive MSSA (27.1 cases/100,000 population) was more than twice that for invasive MRSA (13.1 cases/100,000 population) (p<0.001). Invasive MSSA cases were less likely to be HACO (p<0.001), more likely to be CA (p<0.001), and have no concurrent conditions (p = 0.006) than invasive MRSA cases. Bacteremia (p = 0.026) and pneumonia (p<0.001) were less common among invasive MSSA cases. However, septic arthritis (p<0.001) was more common. Injection drug use and inpatient case-fatality rate were similar for persons with invasive MSSA (6% and 10%, respectively) and invasive MRSA (5% and 11%, respectively) (Table 1).

**Table 1:** Epidemiologic characteristics and concurrent conditions for patients with cases of invasive MSSA and MRSA, Minnesota, USA, August 1, 2014–July 31, 2015*

Characteristic	Total, n = 701				HO, n = 65				HACO, n = 416				CA, n = 220		
	MSSA	MRSA	p value		MSSA	MRSA	p value		MSSA	MRSA	p value		MSSA	MRSA	p value
Total	473 (67)	228 (33)	NA		40 (8)	25 (11)	0.28		260 (55)	156 (68)	<0.001		173 (37)	47 (21)	<0.01
Demographic															
Median age, y	59 (0–102)	62 (0–95)	0.05		61 (0–94)	61 (17–93)	0.99		62 (0–102)	65 (5–95)	0.21		55 (0–90)	50 (0–91)	0.40
Sex															
F	175 (37)	92 (40)	0.39		15 (38)	6 (24)	0.26		108 (42)	66 (42)	0.88		52 (30)	20 (43)	0.11
M	316 (67)	153 (67)	0.94		26 (65)	13 (52)	0.30		169 (65)	109 (70)	0.31		121 (70)	31 (66)	0.60
Private residence 4 d before culture	376 (79)	146 (64)	<0.01		NA	NA	NA		211 (81)	100 (64)	<0.01		165 (95)	46 (98)	0.69
LTCF past year	53 (11)	70 (31)	<0.01		4 (10)	8 (32)	0.05		49 (19)	62 (40)	<0.01		NA	NA	NA
CVC 2 d before culture	29 (6)	19 (8)	0.28		6 (15)	5 (20)	0.74		23 (9)	14 (9)	0.97		NA	NA	NA
Length of stay, d	7 (0–121)	8 (0–58)	0.05		15 (3–121)	15 (4–48)	0.91		6 (0–43)	7 (1–39)	0.11		7 (0–53)	9 (0–58)	0.15
Inpatient death	47 (10)	26 (11)	0.55		8 (20)	2 (8)	0.29		29 (11)	23 (15)	0.28		10 (6)	1 (2)	0.46
Concurrent condition															
None	96 (20)	27 (12)	<0.01		1 (4)	5 (13)	0.39		14 (9)	35 (12)	0.17		12 (26)	56 (12)	0.37
Kidney disease	95 (20)	67 (29)	<0.01		9 (23)	9 (36)	0.24		77 (30)	54 (35)	0.29		9 (5)	4 (9)	0.48
Chronic skin breakdown	22 (5)	25 (11)	<0.01		<0.01	3 (12)	0.69		14 (5)	21 (13)	<0.01		5 (3)	1 (2)	1.00
D/PU	9 (2)	17 (7)	<0.01		0 (0)	0 (0)	NA		6 (2)	17 (11)	<0.01		3 (2)	0 (0)	1.00
CVD	58 (12)	46 (20)	<0.01		5 (13)	6 (24)	0.31		44 (17)	39 (25)	0.05		9 (5)	1 (2)	0.69
H/P	15 (3)	23 (10)	<0.01		1 (3)	2 (8)	0.55		9 (3)	20 (13)	<0.01		5 (3)	1 (2)	1.00
Other drug use†	41 (9)	25 (11)	0.33		4 (10)	2 (8)	1.00		21 (8)	11 (7)	0.70		16 (9)	12 (26)	<0.01
PVD	24 (5)	22 (10)	0.02		3 (8)	4 (16)	0.42		16 (6)	17 (11)	0.08		5 (3)	1 (2)	1.00
COPD	50 (11)	34 (15)	0.10		6 (15)	6 (24)	0.36		30 (12)	23 (15)	0.34		14 (8)	5 (11)	0.57
Current smoker	71 (15)	32 (14)	0.73		7 (18)	4 (16)	1.00		36 (14)	19 (12)	0.63		28 (16)	9 (19)	0.63
Diabetes	153 (32)	80 (35)	0.47		12 (30)	11 (44)	0.25		96 (37)	59 (38)	0.86		45 (26)	10 (21)	0.51
Injection drug use	25 (5)	12 (5)	0.99		0 (0)	0 (0)	NA		10 (4)	4 (3)	0.48		15 (9)	8 (17)	0.11
Obesity	70 (15)	43 (19)	0.17		8 (20)	7 (28)	0.46		47 (18)	29 (19)	0.90		15 (9)	7 (15)	0.27
Syndrome															
Bacteremia	328 (69)	178 (78)	0.03		30 (75)	16 (64)	0.34		181 (70)	125 (80)	0.03		117 (68)	37 (79)	0.14
Internal abscess	25 (5)	11 (5)	0.78		2 (5)	0 (0)	0.52		16 (6)	5 (3)	0.18		7 (4)	6 (13)	0.04
Pneumonia‡	39 (8)	40 (18)	<0.01		2 (5)	8 (32)	<0.01		21 (8)	22 (14)	0.06		16 (9)	10 (21)	0.02
Septic arthritis	137 (29)	35 (15)	<0.01		3 (8)	2 (8)	1.00		67 (26)	24 (15)	0.01		67 (39)	9 (19)	0.01
Skin abscess	7 (1)	4 (2)	0.76		0 (0)	1 (4)	0.39		7 (3)	0 (0)	0.05		0 (0)	3 (6)	<0.01
Bursitis	35 (7)	10 (4)	0.12		0 (0)	1 (4)	0.39		7 (3)	3 (2)	0.75		28 (16)	6 (13)	0.57
Cellulitis	73 (15)	32 (14)	0.60		6 (15)	3 (12)	1.00		38 (15)	18 (11)	0.35		29 (17)	11 (23)	0.30
Endocarditis	23 (5)	9 (4)	0.57		2 (5)	1 (4)	1.00		12 (5)	5 (3)	0.47		9 (5)	3 (6)	0.72
Osteomyelitis	51 (11)	28 (12)	0.58		1 (3)	4 (16)	0.07		38 (15)	20 (13)	0.58		12 (7)	3 (7)	0.75
Septic shock	41 (9)	19 (8)	0.86		3 (8)	4 (16)	0.42		27 (10)	11 (7)	0.24		11 (6)	4 (9)	0.53

*Values are no. (%), median (range), or no. (range). Nonsignificant (p>0.05) differences were observed for chronic pulmonary disease, current smoker, diabetes, intravenous drug use, obesity, bursitis, cellulitis, endocarditis, osteomyelitis, and septic shock among patients with MSSA and MRSA cases and within epidemiologic classifications of MSSA and MRSA cases. CA, community associated; COPD, chronic pulmonary disease; CVC, central venous catheter; CVD, cardiovascular disease; D/PU, decubitus/pressure ulcer; HACO, healthcare-associated, community onset; HO, hospital onset; H/P, hemiplagia/paraplegia; LTCF, long-term care facility; MRSA, methicillin-resistant *Staphylococcus aureus*; MSSA, methicillin-susceptible *S. aureus;* NA, not available; PVD, peripheral vascular disease. †Drug use other than intravenous drug use. ‡All patients with pneumonia had positive blood cultures to meet the case definition.

Isolates from 40% of MSSA cases and 52% of MRSA cases were submitted. Cases with and without submitted isolates did not differ by sex, race, median age, or inpatient case-fatality rate. However, case-patients without isolates were more likely to have invasive MSSA (p = 0.003) or to be hospitalized in the year before culture (p = 0.030).

Invasive MSSA isolates were more genetically diverse than invasive MRSA isolates. We could not assign a USA clonal group for 55% of invasive MSSA isolates compared with 29% of invasive MRSA isolates. The most common clonal group among invasive MSSA isolates was USA200, which included 14% of invasive MSSA isolates; 35% of invasive MRSA isolates were clonal group USA100, and 30% were USA300 (Table 2). The 2 invasive MSSA isolates that were untypable by PFGE were characterized by whole-genome sequencing as sequence type 398; 1 was from a HACO case and 1 was from a hospital onset case.

**Table 2:** Clonal groups and antimicrobial drug susceptibilities of invasive MSSA and MRSA isolates, Minnesota, USA, August 1, 2014–January 31, 2015*

Characteristic	MSSA, no. (%), n = 190	MRSA, no. (%), n = 119	p value
Clonal group			
USA100	13 (7)	42 (35)	<0.01
USA200	26 (14)	0 (0)	<0.01
USA300	5 (3)	36 (30)	<0.01
USA400	1 (1)	3 (3)	0.16
USA500	2 (1)	0 (0)	0.56
USA600	13 (7)	0 (0)	<0.01
USA700	5 (3)	3 (3)	1.00
USA900	10 (5)	0 (0)	<0.01
USA1000	6 (3)	0 (0)	0.09
USA1200	2 (1)	0 (0)	0.56
Untypeable	2 (1)	0 (0)	0.56
No USA group	105 (55)	35 (29)	<0.01
Antimicrobial drug†			
Clindamycin‡	176 (93)	61 (51)	<0.01
Ceftaroline	190 (100)	117 (98)	0.15
Doxycycline	185 (97)	118 (99)	0.41
Erythromycin	143 (75)	18 (15)	<0.01
Gentamicin	189 (99)	118 (99)	1.00
Levofloxacin	175 (92)	35 (29)	<0.01
Penicillin	63 (33)	0 (0)	<0.01
Rifampin	189 (99)	118 (99)	1.00
Tetracycline	177 (93)	118 (99)	0.01
Trimethoprim/sulfamethoxazole	188 (99)	119 (100)	0.53

*MRSA, methicillin-resistant *Staphylococcus aureus*; MSSA, methicillin-susceptible *S. aureus*. †Proportions represent isolates susceptible to an antimicrobial drug. All isolates were susceptible to daptomycin, linezolid, telavancin, and vancomycin. A total of invasive MSSA and MRSA isolates had vancomycin MICs <0.05; 86% of invasive MSSA isolates and 81% of invasive MSSA isolates had vancomycin MICs = 1; 13% of invasive MSSA isolates and 18% of invasive MRSA isolates had vancomycin MICs = 2. ‡Isolates with inducible clindamycin resistance were classified as nonsusceptible.

Invasive MSSA isolates were more often susceptible than invasive MRSA isolates to all antimicrobial drug classes, except for tetracycline (Table 2). We detected penicillin susceptibility through multiple testing methods in 63 (33%) of invasive MSSA isolates. Six clonal groups were identified among penicillin-susceptible isolates.

## Conclusions

The incidence of invasive MSSA was more than twice that of invasive MRSA in these counties. Invasive *S. aureus* infections were associated with a high case-fatality rate. Infection types were similar except for more frequent septic arthritis among invasive MSSA cases and bacteremia and pneumonia among invasive MRSA cases; these findings were consistent with those of other studies (*9*). Some studies found a higher case-fatality rate for MRSA than MSSA infections, possibly attributable to the older age and concurrent conditions among invasive MRSA case-patients (*10*). Although case-patients with invasive MSSA had fewer concurrent conditions and were less likely to have pneumonia (a syndrome associated with poor outcomes [*11*]) than case-patients with invasive MRSA, case-fatality rates were similar.

Invasive MSSA isolates were susceptible to more antimicrobial drugs and were more genetically diverse than invasive MRSA isolates, consistent with results of other reports (*12*). Penicillin susceptibility was observed in 33% of invasive MSSA isolates, which is considerably higher than for previous studies of invasive and noninvasive MSSA isolates and was seen for multiple strain types (*13*,*14*).

Infection control interventions have effectively decreased healthcare-associated invasive MRSA incidence (*15*). However, invasive *S. aureus* burden and mortality rates remain a concern. Most invasive *S. aureus* disease was HACO or CA, highlighting the need for preventing these community-onset infections through new approaches and infection prevention in settings outside acute care. Ongoing surveillance data can inform planning for future interventions, such as improved wound care, enhanced infection prevention in nursing homes and dialysis centers, and greater attention to chronic conditions and development of effective vaccines.
